# Machine learning-based identification and immune characterization of ferroptosis-related molecular clusters in osteoarthritis and validation

**DOI:** 10.18632/aging.205875

**Published:** 2024-05-29

**Authors:** Xiaocheng Guo, Xinyuan Feng, Yue Yang, Wenying An, Lunhao Bai

**Affiliations:** 1 Department of Orthopedics, Shengjing Hospital of China Medical University, Shenyang, China; 2 Department of Cadre Wards, Liaoning University of Traditional Chinese Medicine Affiliated Orthopedic Hospital, Shenyang, China

**Keywords:** osteoarthritis, ferroptosis, differentially expressed genes, immune characteristics, molecular clusters

## Abstract

Osteoarthritis (OA), a degenerative joint disease, involves synovial inflammation, subchondral bone erosion, and cartilage degeneration. Ferroptosis, a regulated non-apoptotic programmed cell death, is associated with various diseases. This study investigates ferroptosis-related molecular subtypes in OA to comprehend underlying mechanisms. The Gene Expression Omnibus datasets GSE206848, GSE55457, GSE55235, GSE77298 and GSE82107 were used utilized. Unsupervised clustering identified the ferroptosis-related gene (FRG) subtypes, and their immune characteristics were assessed. FRG signatures were derived using LASSO and SVM-RFE algorithms, forming models to evaluate OA’s ferroptosis-related immune features. Three FRG clusters were found to be immunologically heterogeneous, with cluster 1 displaying robust immune response. Models identified nine key signature genes via algorithms, demonstrating strong diagnostic and prognostic performance. Finally, qRT-PCR and Western blot validated these genes, offering consistent results. In addition, some of these genes may have implications as new therapeutic targets and can be used to guide clinical applications.

## INTRODUCTION

Osteoarthritis (OA) causes pain and disability in patients and increases the economic burden on the patients and society. The incidence of OA is on the rise owing to the increasing number of elderly and obese individuals worldwide [1, 2]. Recent studies estimate that the overall prevalence of osteoarthritis (hip and knee) is about 300 million worldwide and is the 11th leading cause of disability globally [3]. The etiologic cause of OA has shifted from aging to a multifactorial role, including genetic factors, gender, metabolic syndrome (obesity, hypertension, hyperglycemia, insulin resistance, and dyslipidemia), and diet [4]. In OA, the entire joint is affected by changes in the periarticular muscles, synovium, articular cartilage, ligaments, subchondral bone, and joint capsule [5]. However, deeper insights into the pathogenesis of OA have revealed that this effect is not limited to wear-and-tear and degenerative diseases. The development of OA is complex, entailing both inflammatory and metabolic factors, and the pathogenesis may involve chondrocyte senescence, apoptosis, and active synovitis [6, 7].

As a novel type of programmed cell death that is distinguished from cellular autophagy, apoptosis, and necrosis, ferroptosis (or iron-dependent cell death) triggers the demise of cells by regulating their accumulation of iron-dependent lipid peroxides [8]. Ferroptosis has become a major strategy in the advancement of therapeutic drugs against cancer [9–11] and neurodegenerative, blood, and heart diseases [12]. Moreover, ferroptosis appears at the intersection of infection, inflammation, and immunity [13].

In articular cartilage, water makes up the largest component (>70%), with the remainder (~30%) being the extracellular matrix, which consists mainly of type II collagen and aggrecan. In a pathological state, chondrocytes produce a variety of matrix-degrading enzymes [e.g., a disintegrin and metalloproteinase with thrombospondin-like motifs (ADAMTS)] and inflammatory response proteins (e.g., cytokines) [14]. Yao et al. established a mouse model of OA by surgically introducing interleukin-1 beta (IL-1β) and ferrous ammonium citrate into the animals to mimic inflammation and iron overload, respectively. As a result, the chondrocytes in the OA model mice underwent iron degeneration. In another study, ferritinase-specific inhibitors were shown to decrease the expression of type II collagen and increase matrix metalloproteinase 13 (MMP13) expression in chondrocytes [15, 16]. However, the specific mechanisms underlying the pathology of OA and its relationship with ferroptosis remain unclear.

Microarray and bioinformatics technologies have enabled the extensive genomic, transcriptomic, epigenomic, proteomic, and metabolomic profiling of OA [17–19]. As an interdisciplinary approach [20], bioinformatics based on systems biology and database mining has facilitated a better understanding of the molecular mechanisms behind many diseases [21, 22]. However, the molecular mechanisms underlying the involvement of ferroptosis in the pathogenesis of OA remain unclear. Therefore, further studies are needed to identify new and more reliable therapeutic targets and diagnostic biomarkers to elucidate the specific relationship between ferroptosis and OA.

In this study, differentially expressed genes (DEGs) between samples from patients with OA and healthy controls were identified using gene expression and microarray experiments. Identification of the DEGs and immunophenotyped ferritin-related molecules should help us acquire more in-depth knowledge about the molecular mechanisms of OA.

## MATERIALS AND METHODS

### Microarray dataset search and collection

The GSE206848, GSE55457, GSE55235, GSE77298, and GSE82107 datasets were downloaded from the Gene Expression Omnibus (GEO; http://www.ncbi.nlm.nih.gov/geo) database using the following search terms: (“osteoarthritis”[MeSH Terms] OR [All Fields]) AND “Homo sapiens”[porgn] AND (“gse”[Filter] AND “Expression profiling by array”[Filter]). The GSE206848, GSE55457, GSE55235, and GSE77298 datasets (merged as the training set) contained 34 normal and 68 arthritic tissue samples. The GSE82107 dataset was used as the validation set. The GSE55457 and GSE55235 datasets were sequenced on the GPL96 platform of GEO, whereas the GSE206848 and GSE77298 datasets were sequenced on the GPL570 platform, all with *Homo sapiens* as the reference organism (Supplementary Table 1). The experimental type for all datasets were array expression profiling, as detailed in Supplementary Materials (GEO datasets). The inSilicoMerging package in R/Bioconductor [23] was used to merge the datasets. Furthermore, the methods described by Johnson et al. [24] were used to remove batch effects and obtain the matrix. The FerrDb database [25] was used to identify ferroptosis-related genes (FRGs), whereupon 484 genes were singled out from the gene set after the removal of overlapping genes.

### Identification of differentially expressed ferroptosis-related genes in osteoarthritis

The probes in each dataset were converted into gene symbols according to the platform annotation file. In the case of multiple probes being mapped to the same gene symbol, their average value was used as the gene expression value. In total, expression profiles of 484 FRGs in patients with OA were identified from the FerrDb database (http://www.zhounan.org/ferrdb/current). To obtain the FRGs, the limma package [26] in R was used to perform differential analysis between the OA and control samples, with the following significance thresholds: a *p*-value of less than 0.05 and a fold change of greater than 1.5. Subsequently, secondary gene correlation and functional enrichment analyses were performed on 139 FRGs (49 upregulated and 90 downregulated genes). Volcano and heat maps of the DEGs were drawn using the ggplot2 [27] and pheatmap packages in R, respectively.

### Enrichment analysis

Functional and pathway annotations of the 139 differentially expressed FRGs in OA were performed through Gene Ontology (GO) and Kyoto Encyclopedia of Genes and Genomes (KEGG) enrichment analyses [28], respectively. The main domains in GO analysis are Biological Process (BP), Cellular Component (CC), and Molecular Function (MF). Using the GO and KEGG annotations from the org.Hs.eg.db (version 3.1.0) package in R and KEGG REST API (https://www.kegg.jp/kegg/rest/keggapi.html) as the background, the genes were mapped to the background set and enrichment analysis of the gene set was then performed using the clusterProfiler (version 3.14.3) package [29] in R. The gene set was limited to a minimum of 5 and a maximum of 5000 genes, and a *p*-value of less than 0.05 and a false discovery rate of less than 0.25 were considered statistically significant. Bubble and circle plots were created.

### Machine learning-based screening of key genes and construction of diagnostic models

A least absolute shrinkage and selection operator regression (LASSO) model based on the 139 FRGs was constructed using the glmnet (version 4.1.3) package [30]. The optimal lambda value was used to identify the key genes.

Additionally, the SVM-RFE [31] algorithm with a linear kernel in the caret package of R, combined with 10-fold cross-validation, was used to obtain the optimal variables. Then, the number of genes corresponding to the largest cross-validation accuracy and the smallest error was chosen. The performance of several characteristic variables was evaluated using the RFE function in the caret package. To obtain the input variables for reconstructing the linear SVM model, the most accurate feature variables in the SVM analysis were chosen. Subsequently, the predictive ability of the newly constructed model was tested using the training and validation sets. The area under the receiver operating characteristic (ROC) curve (AUC) values were plotted to assess the diagnostic value of the LASSO and SVM models, using the pROC (version 1.12.1) package [32] in R. By intersecting the results from these two machine learning algorithms, nine key FRGs were finally identified.

### Gene set enrichment analysis

Gene set enrichment analysis (GSEA) can be used to compute gene clusters according to the degree of differential expression between two samples [33]. The nine key genes screened by machine learning were divided into high- and low-expression groups for functional or pathway enrichment analysis. The enrichplot package in R was used to depict the results, and the clusterProfiler package in R was used to analyze the clusters with a p.adj threshold of less than 0.05.

### Analysis of immune infiltration and immune correlation of diagnostic genes

The immune microenvironment comprises mesenchymal stem cells, immune cells, fibroblasts, cytokines, inflammatory cells, and chemokines. Being a synovial joint, the progression of diseases of the knee involves many immune factors, including resident macrophages and fibroblast-like synoviocytes, leukocytes, cytokines, and secreted matrix metalloproteinases [34]. The proportion of various immune cell types in the combined dataset samples was assessed using CIBERSORT [35], following which the Kruskal–Wallis test was used to compare the variability of the distribution of different immune cells between the OA and control groups. Subsequently, the correlation between the expression of diagnostic genes in the constructed models and immune cells with significantly different identifications was calculated using the “cor” function in R.

### Sample subtype and immune correlation analysis based on diagnostic genes

Based on the expression of significant diagnostic genes obtained by screening the combined samples, disease subtype analysis was performed on all OA samples using the ConsensusClusterPlus (version 1.54.0) package [36] in R. Diagnostic gene scores were then evaluated for each sample using the gene set variation analysis (GSVA) (version 1.36.3) package in R [37]. Subsequently, the differences in diagnostic gene scores between the subtypes were compared using the Kruskal–Wallis test. Then, CIBERSORT was used to assess the proportional distribution of the immune cells in the combined samples. The immune and stromal scores of the OA samples in the combined dataset were calculated using the ESTIMATE package [38] in R. The expression of genes coding for immune checkpoint molecules [39] and human leukocyte antigen (HLA) family members was compared between the two groups.

### Culture of SW1353 cell line and real-time PCR (qRT-PCR) reactions

We chose the SW1353 chondrosarcoma cell line to imitate chondrocytes in this study due to the human samples selected for the data set. The human chondrosarcoma cell line SW1353 was maintained at 37° C in 5% CO_2_ in 1% streptomycin, 1% penicillin (Hyclone, USA), 10% FBS (Bioind, Kibbutz Beit-Haemek, Israel) and DMEM (Hyclone). To induce an *in vitro* model of osteoarthritis (OA), SW1353 cells were treated with recombinant human IL-1β (10 ng/ml; Beyotime, China) for 24 h. Here we selected the optimum concentration of 1L-1β as 10ng/ml [40]. Total ribonucleic acid (RNA) was isolated from cell cultures using the RNAiso Plus kit (Vazyme Biotech Co., Ltd., Nanjing, China) according to the manufacturer’s instructions. Next, RNA was reverse transcribed to cDNA using the HiScript III Q RT SuperMix for qPCR (+gDNA wiper; Vazyme Biotech Co., Ltd.). qRT-PCR reactions were prepared using the SYBR Green PCR kit (Vazyme Biotech Co., Ltd.) and Applied Biosystems 7500 Real-Time PCR System. Each reaction was performed in triplicate. PCR conditions were as follows: step 1, 95° C for 30 s; step 2, 95° C for 5 s, 60° C for 20 s, 40 cycles; step 3, 95° C for 15 s, 60° C for 60 s, 95° C for 15 s. Relative mRNA expression was calculated using the 2^-∆∆Cq^ method. The values obtained are represented based on the fold-change relative to GAPDH. The target gene primers were designed and acquired by Sangon, Shanghai, China (Supplementary Table 2). GAPDH was used as internal control.

### Western blot analysis

Chondrocytes in culture plates were washed in cold PBS three times, then lysed in RIPA (9806S, Cell Signaling Technology, USA) with 1 mM PMSF (ST506; Beyotime Biotech, Shanghai, China) and 1 mM phosphatase inhibitors (P1081; Beyotime Biotech, Shanghai, China). The lysates were centrifuged at 12,000 rpm/min for 20 min at 4° C and the supernatants were collected and stored at −80° C. Protein quantification was performed using a BCA Protein Assay Kit (Enhanced) (Beyotime). Equivalent quantities of proteins (30 μg) were separated using polyacrylamide gel electrophoresis (8%–10% SDS-PAGE) and subsequently wet-transferred onto polyvinylidene difluoride (PVDF) membranes. Next, PVDF membranes were blocked with 5% bovine serum albumin (BSA) for 2 h at 25° C and washed thrice using Tris-buffered saline (TBS) with 0.1% Tween-20 (TBST) for 5 min. PVDF membranes were then incubated at 4° C overnight with the following primary antibodies: GABARAPL1-Specific antibody (1:1000; Proteintech, China; 11010-1-AP), Anti-SAT1 antibody (1 μg/ml; Abcam, UK; ab105220), EGF Receptor Antibody (1:1000; Cell Signaling Technology; #2232), Recombinant Anti-ELOVL5 antibody (1:1000; Abcam; ab205535), Anti-NAK/TBK1 antibody (1:5000; Abcam; ab40676), ZIP7 Polyclonal antibody (1:4000; Proteintech; 19429-1-AP), TRIM26 Polyclonal antibody (1:2500; Proteintech; 27013-1-AP), Recombinant Anti-SHP1/TRIM26 antibody (1:1000; Abcam; ab32559), BEX1/2 Polyclonal antibody (1:2500; Proteintech; 12390-1-AP), Anti-Collagen II antibody (1:1000; Abcam; ab34712), MMP13 Polyclonal antibody (1:2000; Proteintech; 18165-1-AP), and GAPDH antibody (1:20000; Proteintech; 10494-1-AP). The next day, PVDF membranes were washed thrice with TBST for 10 min and then incubated with goat anti-rabbit IgG H&L (HRP) (ab6721, 1:10,000, Abcam) for 2 h at 25° C. Membranes were visualized using enhanced chemiluminescence (Millipore, USA). ImageJ software (version: 2.0.0-rc-69/1.52p) was used for quantification. GAPDH was used as internal control.

### Statistical analysis

RStudio (version 5.5.4) was used for all statistical analyses. The Wilcoxon test was used to assess differences between the two groups. Using the corrplot package, Pearson’s correlation analysis was performed to calculate the correlation coefficients between different genes. A two-tailed test result with a *p*-value of less than 0.05 was considered statistically significant.

### Data availability

The original contributions presented in this study are included in the article and Supplementary Material, and further inquiries can be directed to the corresponding author. The datasets generated and/or analyzed during this study are available in the Gene Expression Omnibus (GEO) Dataset (http://www.ncbi.nlm.nih.gov/geo/).

## RESULTS

### Analysis and identification of differentially expressed ferroptosis-related genes

**Figure 1:**
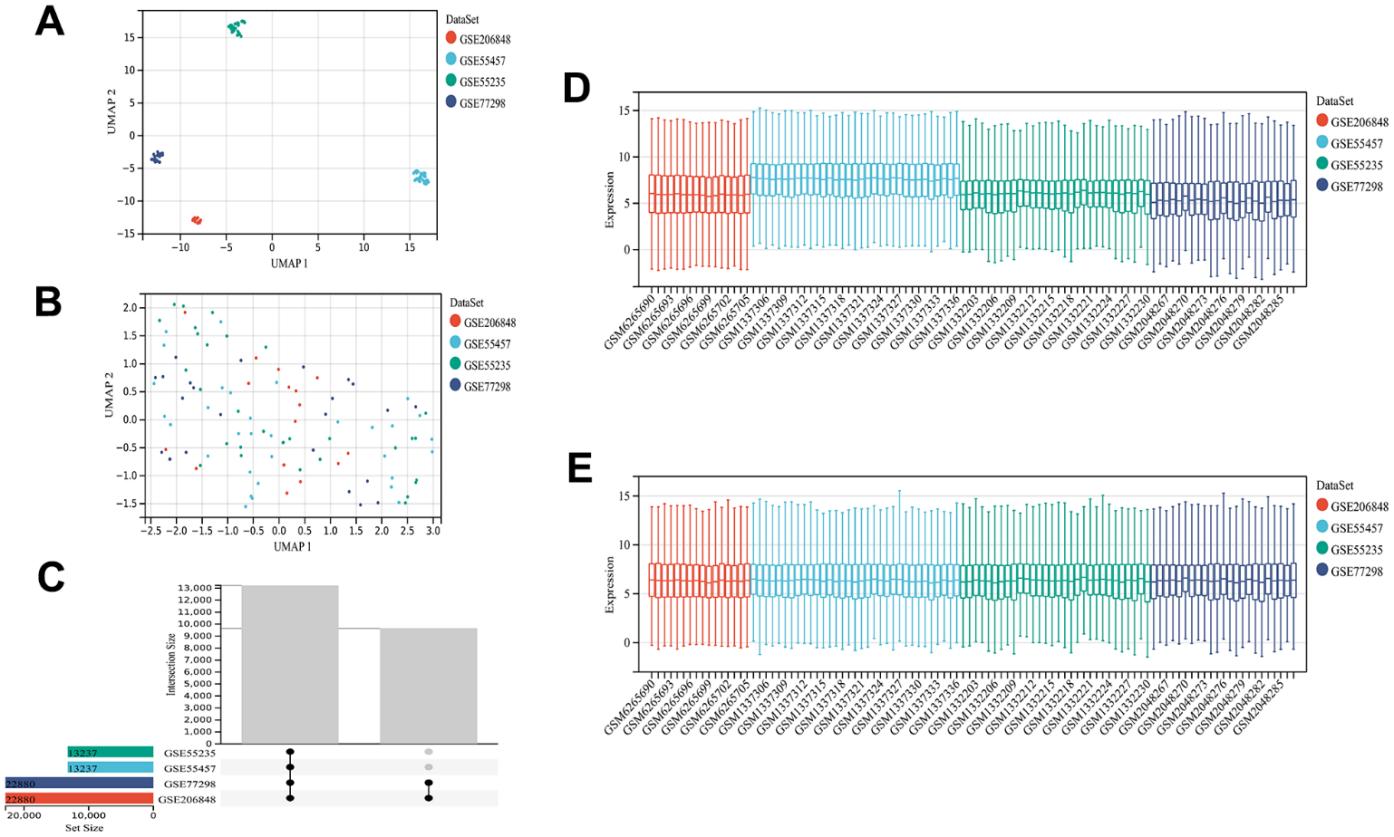
**Dataset information and merging.** UMAP plot before de-batching effect (**A**) and UMAP diagram after de-batching (**B**). Sample content of each dataset (**C**). Box plot before (**D**) and after removing the batch effect (**E**). The data distribution tends to be consistent among the data sets after removing the batch effect, and the median is on a line. The samples of each dataset after removing the batch effect are clustered and intertwined with each other, suggesting a better removal of the batch effect.

**Figure 2:**
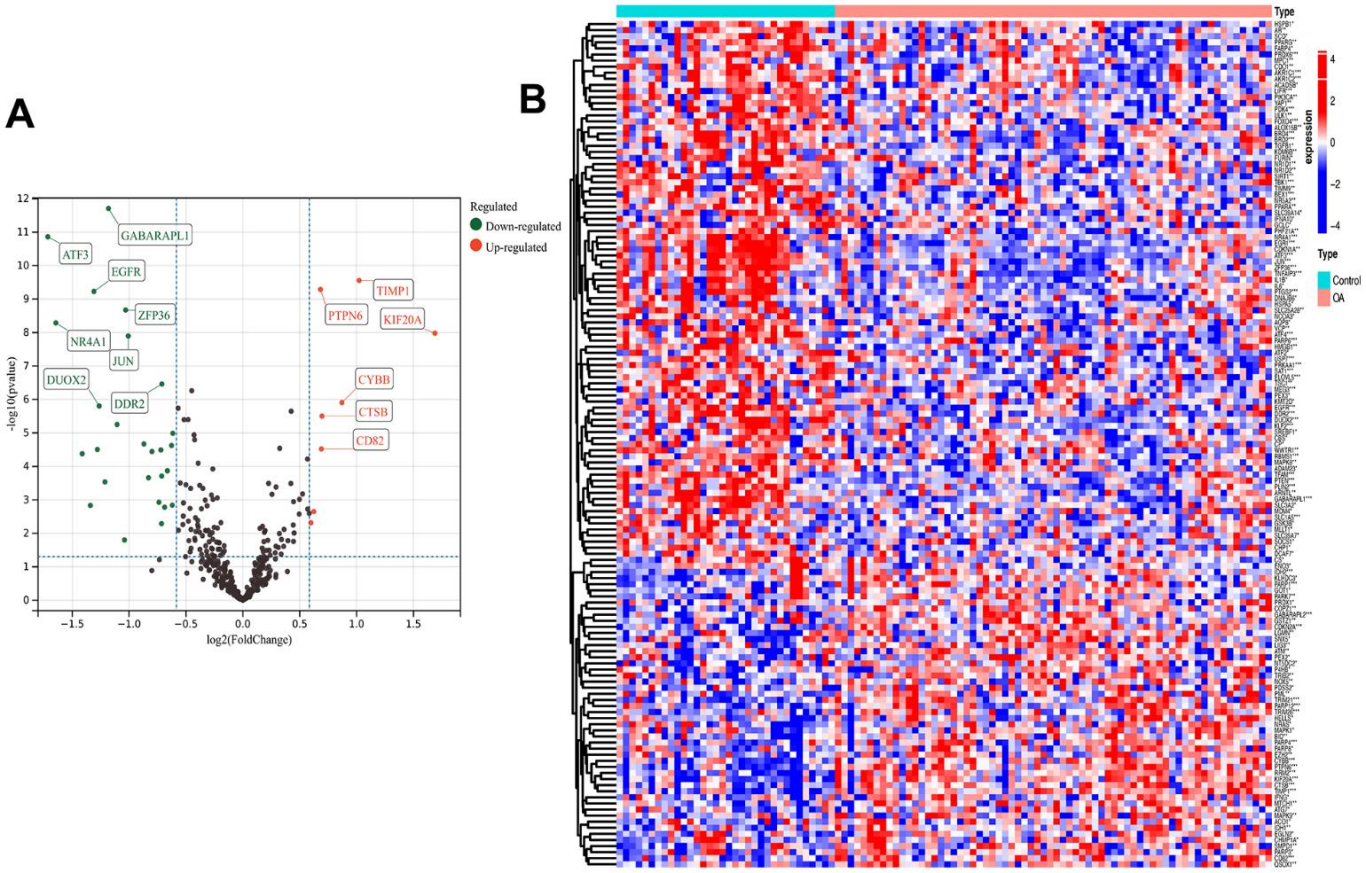
**Differential analysis.** Ferroptosis-related differentially expressed genes (FRGs) volcano plot (**A**) with log2FoldChange in the horizontal coordinate and -log10(P-value) in the vertical coordinate. Red nodes indicate upregulated FRGs, green nodes indicate downregulated FRGs, and black nodes indicate genes that are not significantly differentially expressed. Heat map (**B**) of ferroptosis-related DEG expression levels: p-value<0.001: “***”, p-value<0.01: “**”, p-value<0.05: “*”.

The analytical process used in this study is shown in Supplementary Figure 1. The GEO datasets were corrected for batch effects, yielding 68 OA and 34 control samples for analysis. Then, the usability of the combined matrix was evaluated as shown in the box and density plots (Figure 1A, 1B). The sample size and integration of the dataset are shown in Figure 1C. After elimination of the batch effects, the data distribution was consistent among each dataset, with the median appearing on a straight line and similar means and variances (Figure 1D, 1E). The expression of 484 FRGs were subsequently obtained using the integrated dataset. The limma package identification of FRGs that were differentially expressed between the OA and control samples yielded 49 upregulated and 90 downregulated genes (Figure 2A). Figure 2B shows a heat map of the 139 FRGs and their normalized expression.

### GO functional and KEGG pathway analyses of the differentially expressed ferroptosis-related genes

**Figure 3:**
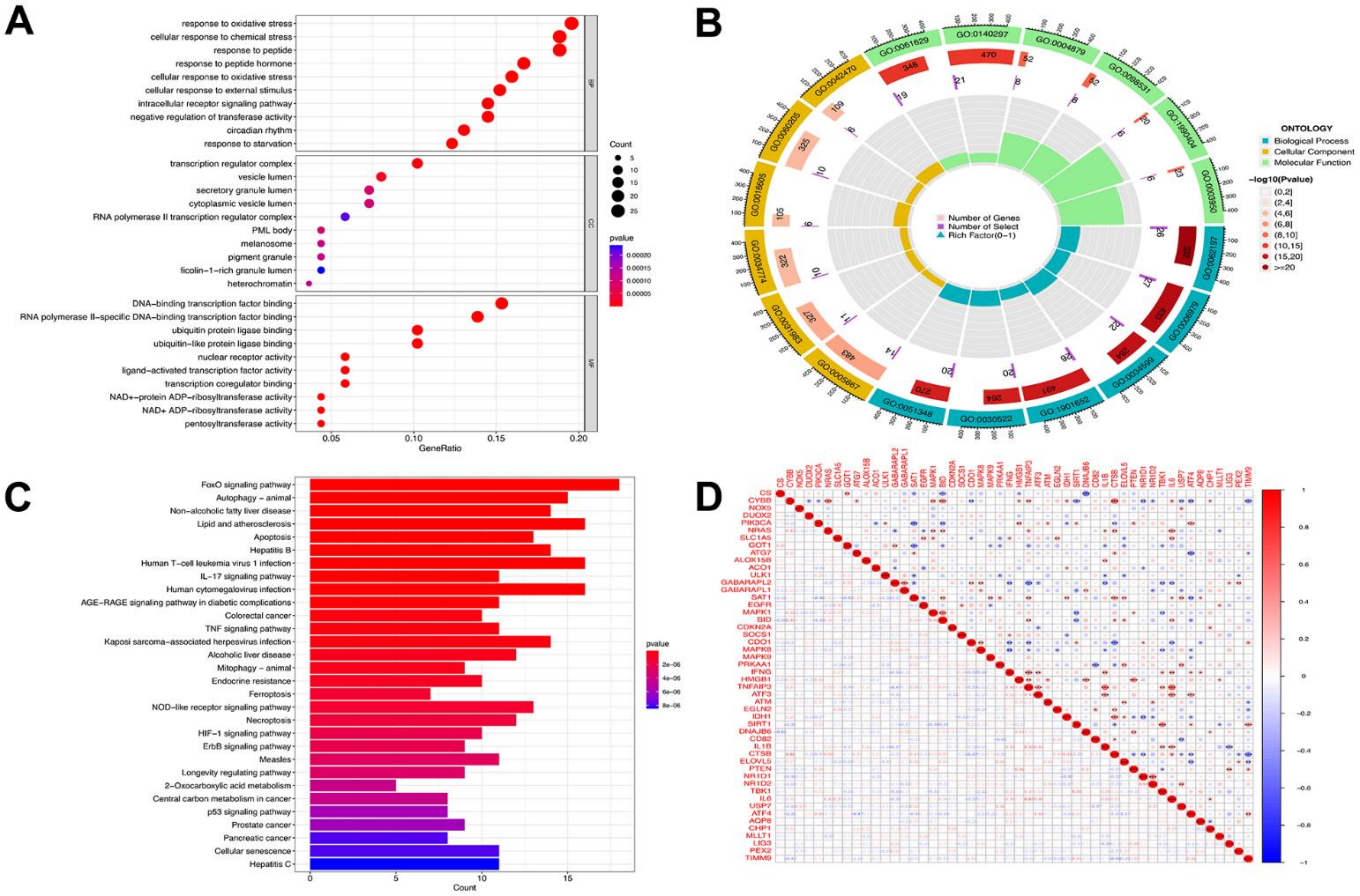
**Functional analyses for the DE-FRGs.** Bubble plot (**A**) of functional enrichment analysis including molecular functions (MF), cellular components (CC), and biological processes (BP), and circle plot (**B**) display. Results of the KEGG pathway analyses of the DE-FRGs with barplot (**C**). Differential genes correlation heat maps (**D**) plotted using the corrplot package, we found GABARAPL2 had a negative correlation with TNFAIP3, IL1B, TBK1, and IL6. TNFAIP3 was positively correlated with ATF3, IL1B, TBK1 and IL6. Blue represents a negative correlation, and red represents a positive correlation.

Enrichment analysis was performed to investigate which biological processes the 139 genes were involved in. The genes were significantly enriched in the cellular response to oxidative stress, transcription regulator complex, ubiquitin protein ligase binding, and RNA polymerase II-specific DNA-binding transcription factor binding terms of the GO MF category; the cellular response to chemical stress and response to peptide terms of the BP category; and the DNA-binding transcription factor binding, vesicle lumen, and secretory granule lumen terms of the CC category (Figure 3A, 3B). The KEGG analysis revealed that these genes were mainly involved in the ferroptosis, autophagy, apoptosis, AGE-RAGE signaling in diabetic complications, FoxO signaling, interleukin-17 (IL-17) signaling, and tumor-necrosis factor (TNF) signaling pathways (Figure 3C). Spearman’s correlation analysis revealed that GABA type A receptor associated protein like 2 (*GABARAPL2*) gene expression was negatively correlated with TNF alpha induced protein 3 (*TNFAIP3*), *IL1B*, TANK binding kinase 1 (*TBK1*), and interleukin-6 (*IL6*) gene expression, whereas the expression of *TNFAIP3* was positively correlated with that of activating transcription factor 3 (*ATF3*), *IL1B*, *TBK1*, and *IL6* (Figure 3D).

### Construction and validation of the ferroptosis prediction model

**Figure 4:**
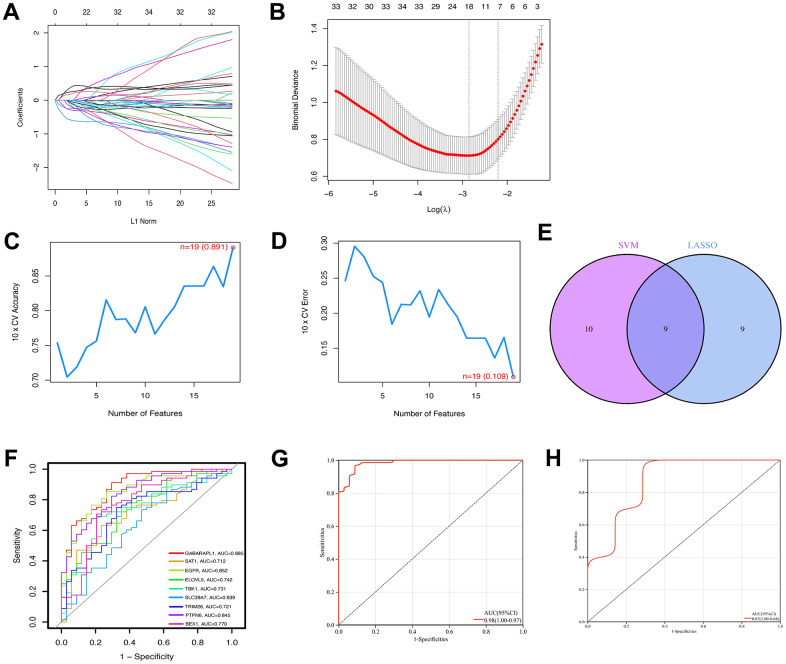
**Construction of ferroptosis signature using machine learning algorithms.** The LASSO coefficient profiles of the 9 co-expressional ferroptosis genes (**A**). The optimal lambda value was selected in the LASSO regression model based on 10-fold cross-validation (**B**). The line graph shows the cross-validated accuracy based on different numbers of ferroptosis genes in the SVM-RFE model (C and D). The 19 genes with the highest cross-validation accuracy (**C**) and the lowest error (**D**) were selected. Screening of nine key ferroptosis genes (**E**) using LASSO and SVM-RFE machine learning algorithms. ROC curves for the 9 marker genes (**F**). Logistic regression model to identify the AUC (**G**) of ferroptosis-related osteoarthritis samples. Model validation of disease diagnosis models in the independent validation dataset GSE82107 (**H**).

Of the 139 FRGs, 18 were found by the LASSO algorithm to have remarkable effects on arthritis (Figure 4A, 4B), whereas the SVM-RFE algorithm identified 19 effective predictors (Figure 4C, 4D). Intersection of the results of the two algorithms revealed nine common ferroptosis-related predictive genes: *GABARAPL1*, spermidine/spermine N1-acetyltransferase 1 (*SAT1*), epidermal growth factor receptor (*EGFR*), ELOVL fatty acid elongase 5 (*ELOVL5*), *TBK1*, solute carrier family 39 member 7 (*SLC39A7*), tripartite motif containing 26 (*TRIM26*), protein tyrosine phosphatase non-receptor type 6 (*PTPN6*), and brain expressed X-linked 1 (*BEX1*) (Figure 4E). Additionally, the risk scores were calculated on the basis of the coefficients of the diagnostic genes, the results of which are detailed in Supplementary File 1 (LASSO coefficients). The effectiveness of the disease diagnostic model was tested with the combined training set and the validation dataset, using the ROC curve analysis method (Figure 4G, 4H.). As shown in Figure 4F, the efficiency of the nine-gene signature in predicting OA was good, with the diagnostic model performing well for both the training (AUC = 0.98) and validation (AUC = 0.87) sets. The expression data and risk scores of the samples are shown in Supplementary Files 2, 3.

### Gene set enrichment analysis of the screened key genes and immune analysis

**Figure 5:**
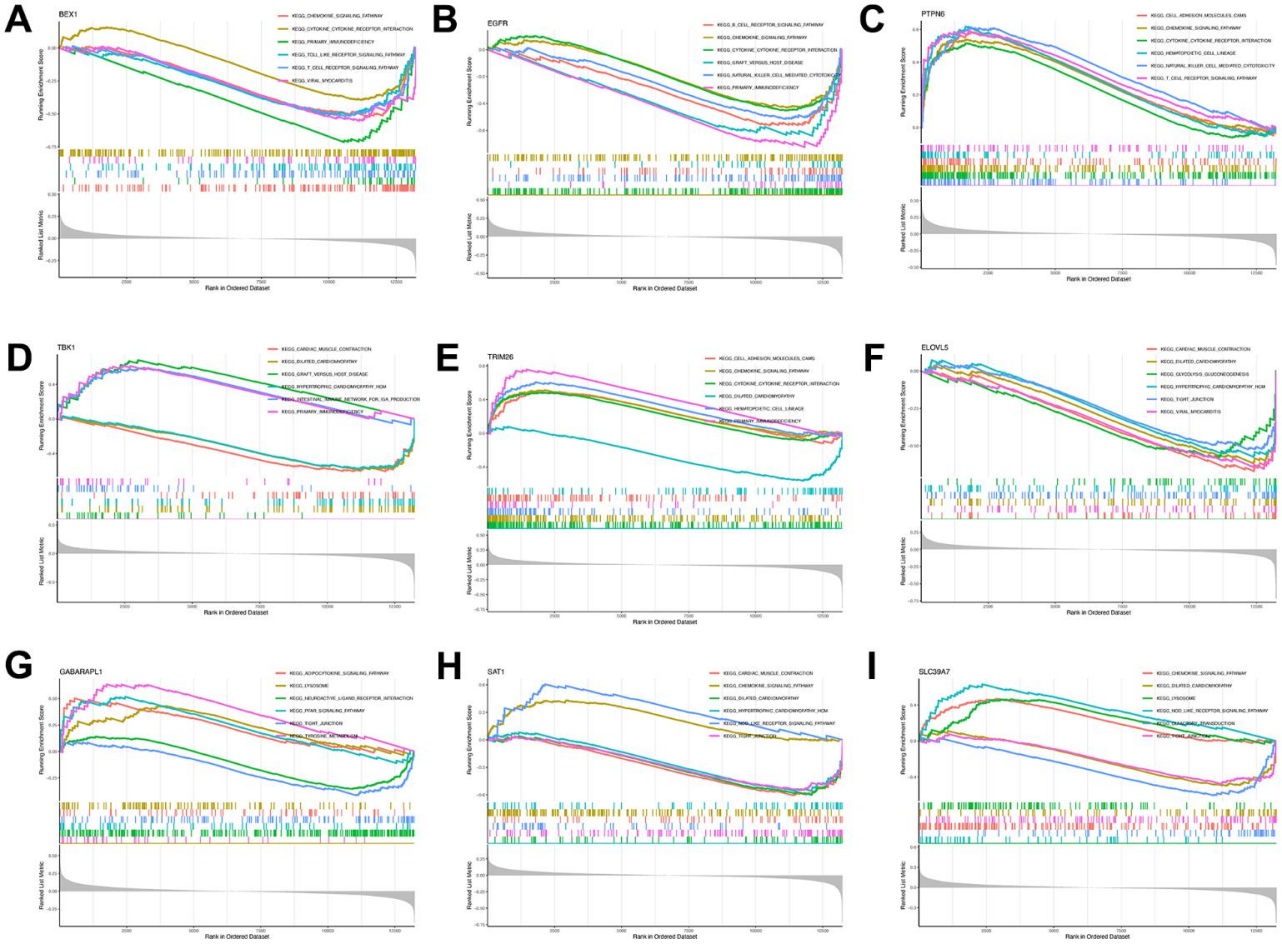
**Single-gene GSEA-KEGG pathway analysis.** BEX1 (**A**), EGFR (**B**), PTPN6 (**C**), TBK1 (**D**), TR1M26 (**E**), ELOVL5 (**F**), GABARAPL1 (**G**), SAT1 (**H**) and SLC39A7 (**I**).

**Figure 6:**
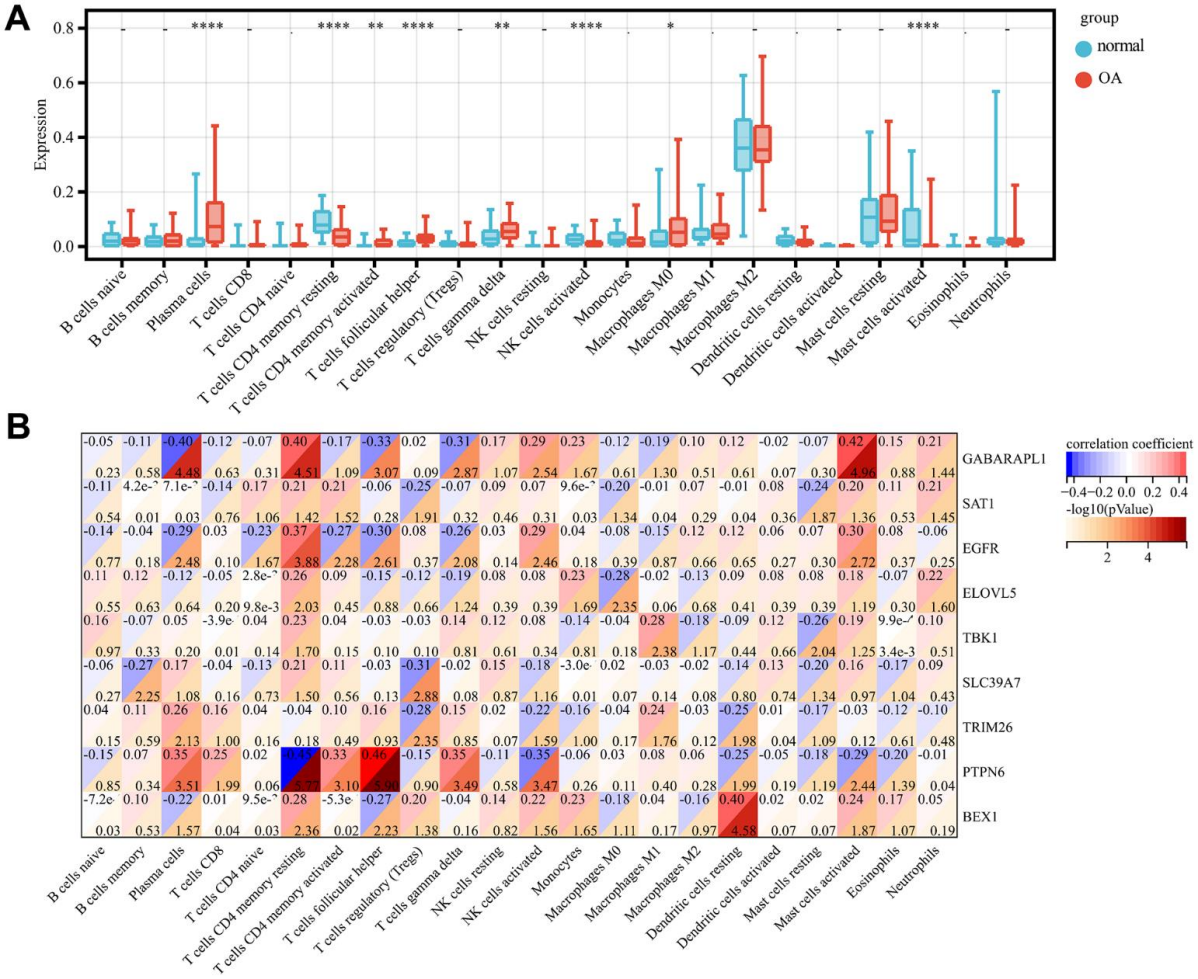
**Immune infiltration analysis.** Comparison of the proportion of 22 immune cell types in OA and normal samples (**A**). Heat map display of 9 diagnostic genes with immune cell correlation (**B**). p-value<0.0001: “****”, p-value<0.001: “***”, p-value<0.01: “**”, p-value<0.05: “*”.

GSEA-KEGG pathway analysis was conducted to explore the pathways in which the nine key genes were enriched in the high- and low-expression clusters. The top six pathways enriched in each key gene are illustrated in Figure 5A–5I. The enriched pathways were mainly related to chemokines, cytokines, cytokine receptors, T-cell receptors, Toll-like receptors, classic signaling, B-cell receptors, cell adhesion molecules, natural killer cell-mediated cytotoxicity, and immunodeficiency. These results suggest that changes in immune cells may be related to OA progression [41]. Therefore, the proportions of 22 types of immune cells between the two groups were compared using the Kruskal–Wallis test (Figure 6A), whereupon eight immune cell types displayed significant differences; namely, M0 macrophages, follicular helper T cells, activated mast cells, plasma cells, activated CD4 memory T cells, resting CD4 memory T cells, gamma-delta T cells, and activated natural killer cells. Furthermore, heatmap of immune cell correlations (Figure 6B) between the nine key genes used to construct the diagnostic model and the immune cells were evaluated (Supplementary File 4). The largest positive correlation was between *PTPN6* and follicular helper T cells (correlation coefficient: 5.90), whereas the largest negative correlation was between resting CD4 memory T cells and *PTPN6* (correlation coefficient: –0.45). These results imply that OA progression is associated with alterations in the immune system.

### Correlation analysis of osteoarthritis phenotypes and subtypes with immunity using unsupervised clustering

**Figure 7:**
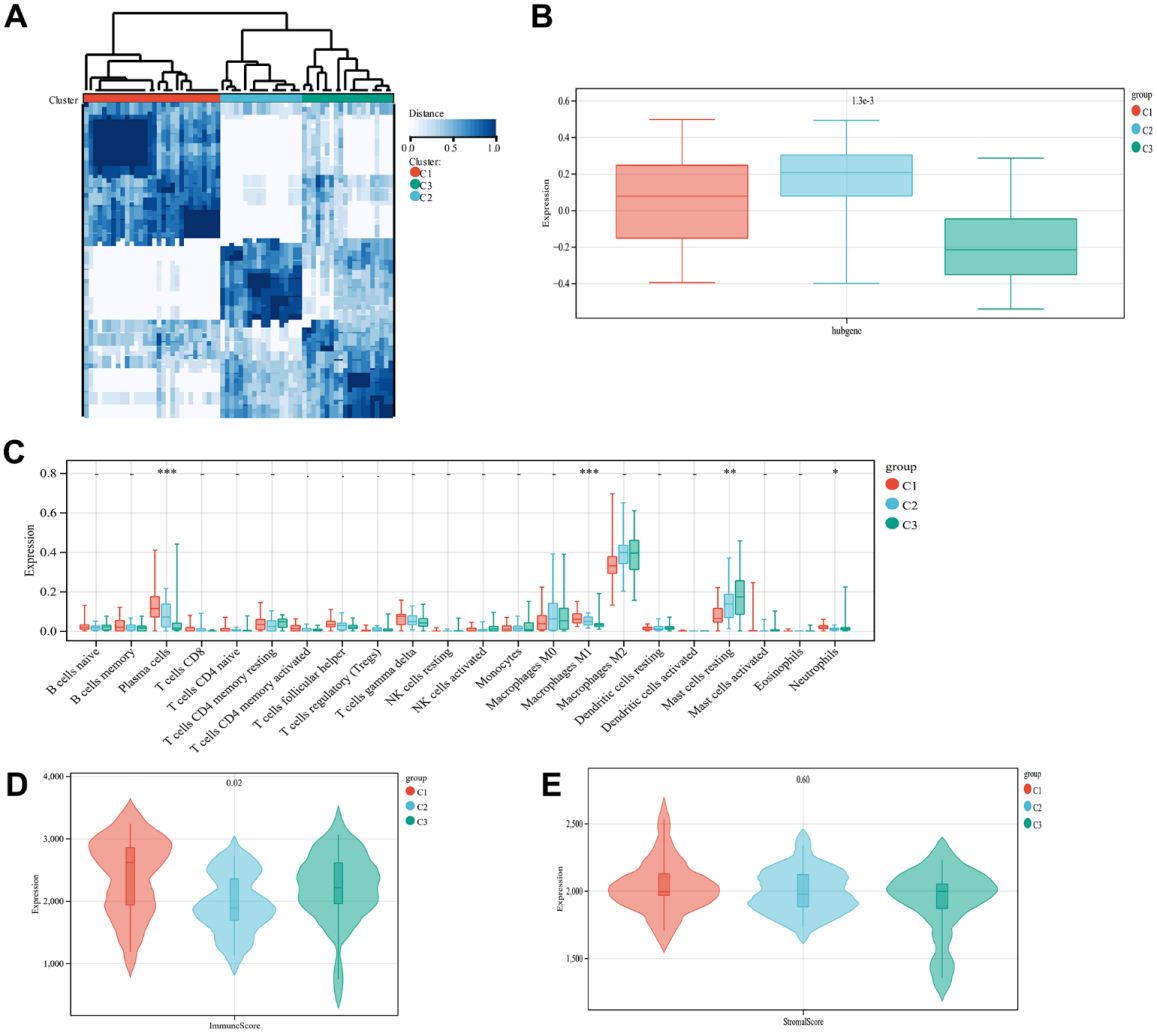
**Unsupervised cluster analysis.** Sample subtype analysis clustering chart (**A**). Comparison of diagnostic gene scores between different subtype groups (**B**). Showing the distribution of various types of immune cells in samples from different subtype groups (**C**). The distribution of immune scores in different subtype groups of samples is displayed (**D**). The distribution of stromal scores in the samples of different subtype groups is displayed (**E**).

Based on the expression of the diagnostic genes, OA subtype analysis was performed on all 68 patient samples, whereupon three different and significant subtype clusters were obtained; namely, C1, C2, and C3 comprising 30, 18, and 20 OA samples, respectively (Figure 7A). The attribution information of each sample is shown in Supplementary File 5 (unsupervised clustering subtype distribution). Subsequently, the GSVA algorithm was used to evaluate the diagnostic gene scores of each cluster, the results of which are detailed in Supplementary File 6 (GSVA scores). The differences in diagnostic gene scores among the different subtype groupings are shown in Figure 7B. The proportion of immune cells in the combined samples was again assessed using CIBERSORT, following which the immune and stromal scores for the OA samples in the combined dataset were assessed, as presented in Supplementary File 7 (immune scores). Then, the Kolmogorov–Smirnov test was used to assess the differences in the distribution of individual immune cell proportions and estimate scores in the different subtype clusters. Four immune cell types (plasma cells, M1 macrophages, resting mast cells, and neutrophils) with significantly different distributions among the different subtype clusters were screened. The differences in immune scores between the three clusters were significant but not so for the stromal scores (Figure 7C–7E).

The expression of the immune checkpoint and HLA family genes was extracted from the combined data samples, and the variability in expression between the different subtype clusters was compared using the Kolmogorov–Smirnov test (Supplementary Figure 2A, 2B). Sixteen HLA family genes as well as four immune checkpoint genes (*CD27*, *CD86*, *CTLA4*, and *IDO1*, coding for CD27 protein, CD8 protein, cytotoxic T-lymphocyte associated protein 4, and indoleamine 2,3-dioxygenase 1, respectively) were identified that differed significantly among C1, C2, and C3. Analysis of the immune molecular clusters in ferroptosis-associated OA suggests that immune checkpoint molecules with significant expression differences may play a key role in the development of this joint disease.

### Validation of key genes in IL-1β induced chondrocyte inflammation and inflammation-related protein/mRNA expression

**Figure 8:**
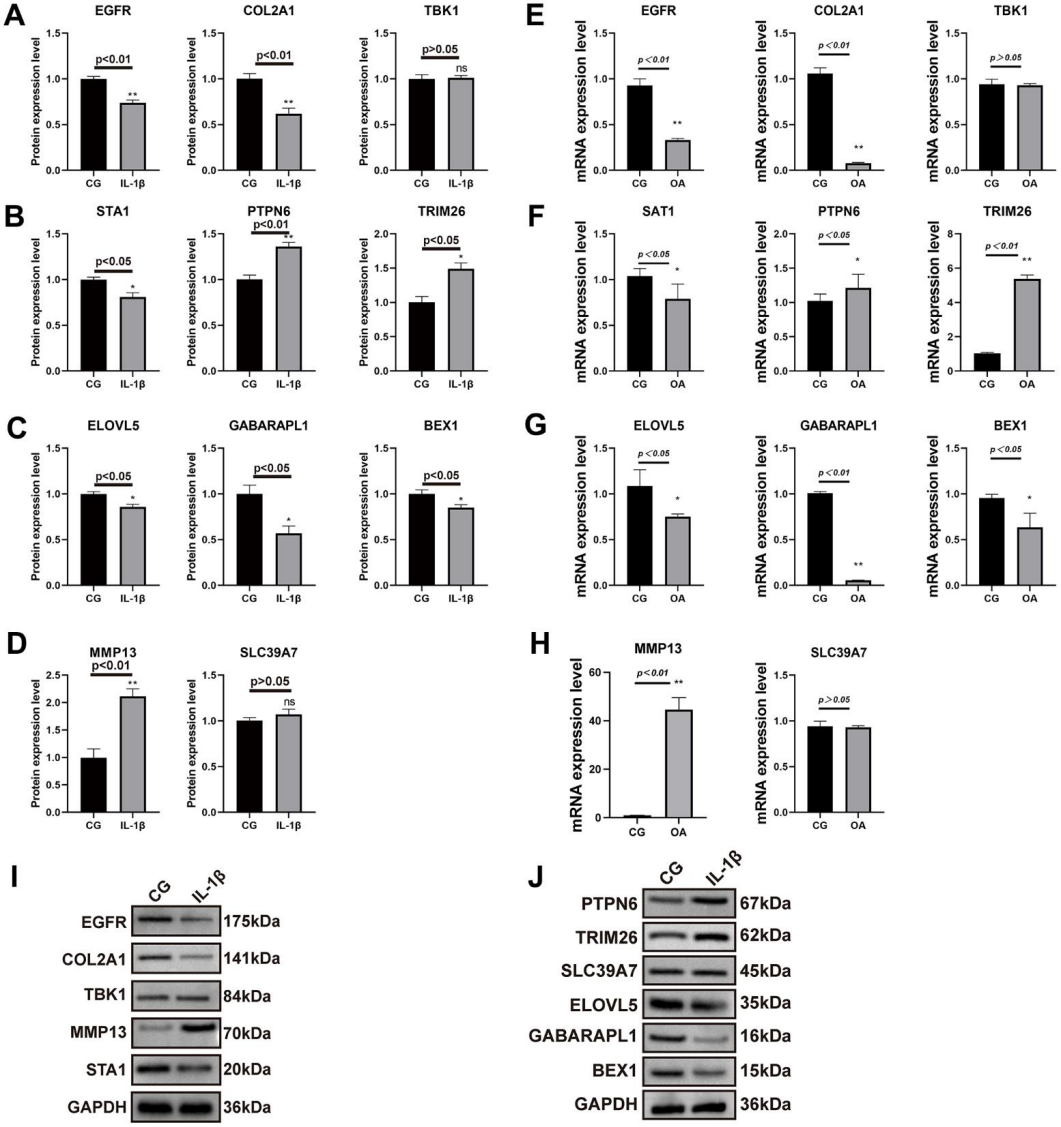
**Results of real-time PCR and Western blot analysis.** The SW1353 cell line was divided into two groups: normal chondrocytes and those with the addition of interleukin 1β (10ng/ml) intervention. The results of COL2A1 and MMP13 with GAPDH as an internal control (**A**–**D**) and real-time PCR was performed to measure the relative mRNA expression of inflammatory genes/proteins in OA (**E**–**H**). Western blotting analysis of nine key genes in two groups (**I**, **J**).

To further validate the expression of key genes for constructing the model on OA chondrocytes, we performed qRT-PCR and Western blot analyses. We first detected the relative mRNA expression of disease-related factors in the CG and 1L-1β (10ng/ml) groups. As shown in Figure 8E–8H, IL-1β-treated chondrocytes showed upregulations of MMP-13 mRNA, whereas a downregulation of collagen II mRNA. This simulates the environment of OA *in vitro*. In addition, we did Western blot and qRT-PCR validation for each of the nine key genes screened by the machine learning methods described above, and the results obtained were almost consistent with our predictions (Figure 8A–8D, 8I, 8J).

## DISCUSSION

With increasing insights into the development of OA, the treatment options for the condition have focused on pain relief through methods such as physical activity, pharmacologic, surgical, and complementary or alternative interventions (or both), nonpharmacologic treatment, patient education, weight control, and therapeutic exercise, which are typically used in combination to achieve optimal results. As understanding of OA development grows, treatment strategies emphasize pain relief through a combination of physical activity, medication, surgery, complementary or alternative interventions, nonpharmacologic approaches, patient education, weight management, and therapeutic exercise to achieve optimal results [42, 43]. One study showed that it is important for patients to perform adequately dosed and progressive exercises (e.g., frequency and intensity) [44]. Recent studies have shown that abnormal bone remodeling of the subchondral bone may occur early in OA, which in turn leads to changes in synovial tissue, and synovitis with high levels of macrophages has been found in later stages of OA [45]. Inflammation is accompanied by activation of the immune system, and a study by Hu et al. showed differences in the infiltration of multiple immune cells between osteoarthritic and normal tissues [36]. In the OA synovium, the predominant cells are T-lymphocytes and macrophages, which together with activated synoviocytes promote cytokine production and angiogenesis, leading to a vicious cycle that ultimately results in permanent cartilage degeneration [46]. Because OA is a multifactorial and heterogeneous disease, variations in OA immune cells and their activity lead to loss of immune homeostasis, and the choice of treatment has extended to the cellular level, including intraarticular injections of mesenchymal stem cells [47] and extracellular vesicles [48]. Therefore, deepening our understanding of OA at the immune molecular level has important implications for individualized treatment.

In 2012, scientists proposed a new type of cell death called ferroptosis, a phenomenon in which iron-dependent lipid peroxidation in the cell leads to cell death [49, 50]. Ferroptosis is classified as a form of regulatory necrosis, which is more related to immunity than apoptosis, and compelling evidence suggests that the process also plays a key role in inflammation [51]. The main promoter of iron toxicity is lipoxygenase, an iron-containing enzyme that is dependent on the activation of acyl-CoA synthetase long chain family member 4 (ACSL4)-dependent lipid synthesis. The inhibitor of iron toxicity is thought to be glutathione peroxidase 4 (GPX4), a selenium-containing enzyme that produces glutathione by activating solute carrier family 7 member 11 (SLC7A11). Inactivation of the Xc¯–glutathione (GSH)–GPX4 axis-dependent antioxidative defense system leads to the accumulation of lipid hydroperoxides and ultimately iron toxicity [52]. Phospholipid hydroperoxides play a role in the synthesis and activation of polyunsaturated fatty acids, as enforcers of iron toxicity [53]. Furthermore, ferroptosis is associated with many pathways, including the GPX4-independent surveillance, E-cadherin–NF2–Hippo–YAP, 5′-AMP-activated protein kinase (AMPK) signaling, and hypoxia signaling pathways, all of which regulate iron toxicity. Specifically, ferroptosis regulates intracellular Fe^2+^ metabolism and reactive oxygen species production [54]. Although the association between ferroptosis and disease is becoming increasingly apparent, the molecular mechanisms through which it affects OA or whether it contributes to the disease progression at all remain unclear. Therefore, investigating the potential mechanisms of posting intoxication in OA and disease subtypes associated with iron toxicity is necessary. In this study, a systematic analysis of FRG expression profiles in normal and OA samples was performed. A total of 139 differentially expressed FRGs between the two groups of samples was identified, suggesting that OA may be associated with ferroptosis. To investigate how the relationship of regulators differed between the two samples, the correlation between regulators of ferroptosis was calculated. Furthermore, the changes in the proportions of immune cells were investigated which revealed that patients with OA had higher levels of gamma-delta T cells, plasma cells, follicular helper T cells, activated CD4 memory T cells, and M0 macrophages, in agreement with the findings of previous studies [55]. Functional and pathway enrichment analyses of the differentially expressed FRGs revealed that they were involved in DNA replication, transcription, protein biosynthesis, metabolism, immune regulation, and response to stress. Additionally, the LASSO and SVM-RFE algorithms identified nine key genes, which were subsequently revealed by the GSEA algorithm as being enriched in pathways related to cytokines, cytokine receptors, T and B cell receptors, Toll-like receptors, classic signaling, and immunodeficiency in the high- and low-expression groups. Studies have proven that there is a strong correlation between ferroptosis and the immune response [56–58]. T cells with GPX4 defects accelerate the accumulation of membrane lipid peroxides and to trigger cellular iron toxicity [59]. Moreover, the increase in mitochondrial superoxide and IL-1β levels in GPX4-deficient Treg cells enhances the T helper 17 cell responses [60]. Ferroptosis activation plays a vital role in driving B-cell and natural killer cell differentiation through the suppression of bone morphogenetic protein [13]. Thus, the relationships between the three FRG clusters and infiltrated immune cells were investigated. Cluster 1 showed strong association with activated plasma cells and neutrophils, while cluster 2 correlated with activated plasma cells and M1 macrophages, and cluster 3 associated predominantly with activated mast cells, M2 macrophages, and neutrophils. The distinction in immune scores between the different subtype groups was remarkable but not so for the stromal scores. Activated neutrophils and mast cells are important elements in the initiation and progression of various diseases and are involved in autophagy [61–63]. Interferon-gamma and lipopolysaccharide induce macrophage polarization into the M1 subtype, whereas IL-4 stimulates formation of the M2 subtype. As a result, macrophages have different metabolic profiles and are involved in many signaling pathways, such as those of peroxisome proliferator-activated receptors, c-Myc, hypoxia-inducible factors, phosphoinositide-3-kinase–protein kinase B (PI3K-AKT), and AMPK [64]. Additionally, the patients in cluster 1 had higher immune scores, revealing that these patients had a worse disease prognosis, which was related to an increased immune response. Immune cells can be attracted to the amino acid oxidation products released by iron-toxic cells, resulting in a different distribution among the three groups of patients [65]. Iron uptake disorder may be associated with different OA subtypes; therefore, a model constructed from the 139 FRGs was used to identify genes that may play a key role in the disease. To determine the OA subtypes, a diagnostic model was constructed using the nine key genes (*GABARAPL1*, *SAT1*, *EGFR*, *ELOVL5*, *TBK1*, *SLC39A7*, *TRIM26*, *PTPN6*, and *BEX1*) identified by the LASSO and SVM-RFE algorithms.

GABARAPL1 was identified as an estrogen-regulated early gene. GABARAP promotes the polymerization of tubulin by interacting with GABA type A receptor and tubulin [66]. Moreover, silencing GABARAPL1 impairs the secretion of extracellular vesicles, which have proangiogenic properties [67]. In a study on the molecular mechanism of OA in relation to the theory of autophagy, it was shown that GABARAPL1 downregulated among OA synovial tissues compared with non-OA synovial tissues, which is consistent with our results [68]. SAT1 is the rate-limiting enzyme for the conversion of spermidine and spermine to putrescine. P53 can promote ironosis by repressing the expression of SLC7A11 or upregulating SAT1 and glutaminase 2 (GLS2) expression [69, 70]. EGFR is a key factor in determining whether cellular autophagy is toxic [71]. Yulong Wei et al. showed that EGFR, a cartilage-specific epidermal growth factor receptor, accelerates knee joint deterioration in mice deficient in it [72]. And our study also verified its decreased mRNA levels in the OA group. ELOVL catalyzes fatty acid elongation and also affects cell proliferation and invasion [73]. A prostate cancer study showed that depletion of ELOVL5 altered mitochondrial morphology and function, leading to reactive oxygen species production, and that supplementation of ELOVL5 direct products reversed oxidative stress [74]. TBK1 drives autoinflammation through the regulation of IFN-I, nuclear factor-kappa B (NF-κB), and TNF-induced receptor-interacting serine/threonine-protein kinase 1 (RIPK1)-dependent cell death [75]. SLC39A7 (ZIP7), a zinc transporter family member, is a novel determinant of ferroptosis, consistent with our study findings [76]. Consistent with our validation results those members of the SLC39a family transport zinc from the extracellular or intracellular endoplasmic reticulum (ER) to the cytoplasm, a decrease in the SLC39A7 gene resulted in increased ER zinc levels, impaired cell proliferation, and endoplasmic reticulum stress onset [77]. A study using TRIM26 knockout mice inhibited downstream kinase activation, resulting in reduced induction of pro-inflammatory factors following LPS, TNF-α and 1L-1β stimulation [78], corroborating the increased TRIM26 expression in our cellular model. TRIM26, PTPN6 (a cytoplasmic phosphatase), and BEX1 play critical roles in tumor immune inflammation [78–80]. Interestingly, the validation results for TBK1 and PTPN6 did not show significant differences. Consistently, the patients in cluster 1 had higher immune scores than those in clusters 2 and 3. A high AUC of the model was observed for both the validation and training datasets. More importantly, immune checkpoint and HLA family genes were extracted from the three clusters and found differences among them. Our results highlight the need for subsequent investigations of ferroptosis-related immune checkpoint molecules in OA. This study had some limitations, the primary one of which was the lack of additional clinical features, including OA-related assessment scales, for validating the predictive performance of the model. Future *in vivo* and *in vitro* studies are needed to validate these diagnostic genes, which could be significant for achieving precise treatment of OA.

## CONCLUSIONS

In this study pertaining to OA, significant prognostic and immunologic differences were found among the three identified clusters with ferroptosis-related immunity. The OA prediction model, constructed using nine key genes identified by the LASSO and SVM-RFE algorithms, had good diagnostic value and a strong ability to classify the molecular subtypes of OA. This study provides a theoretical basis for future prognostic and pathological studies on patients with OA.

## Supplementary Material

Supplementary Figures

Supplementary Tables

Supplementary File 1

Supplementary File 2

Supplementary File 3

Supplementary File 4

Supplementary File 5

Supplementary File 6

Supplementary File 7
