# Supplementary figures and images for: Machine learning-based identification and immune characterization of ferroptosis-related molecular clusters in osteoarthritis and validation
Source: Aging (Albany NY). 2024 May 29;16(11):9437–59. doi: 10.18632/aging.205875 (PMC11210262; doi:10.18632/aging.205875)

## SUPPLEMENTARY FIGURES

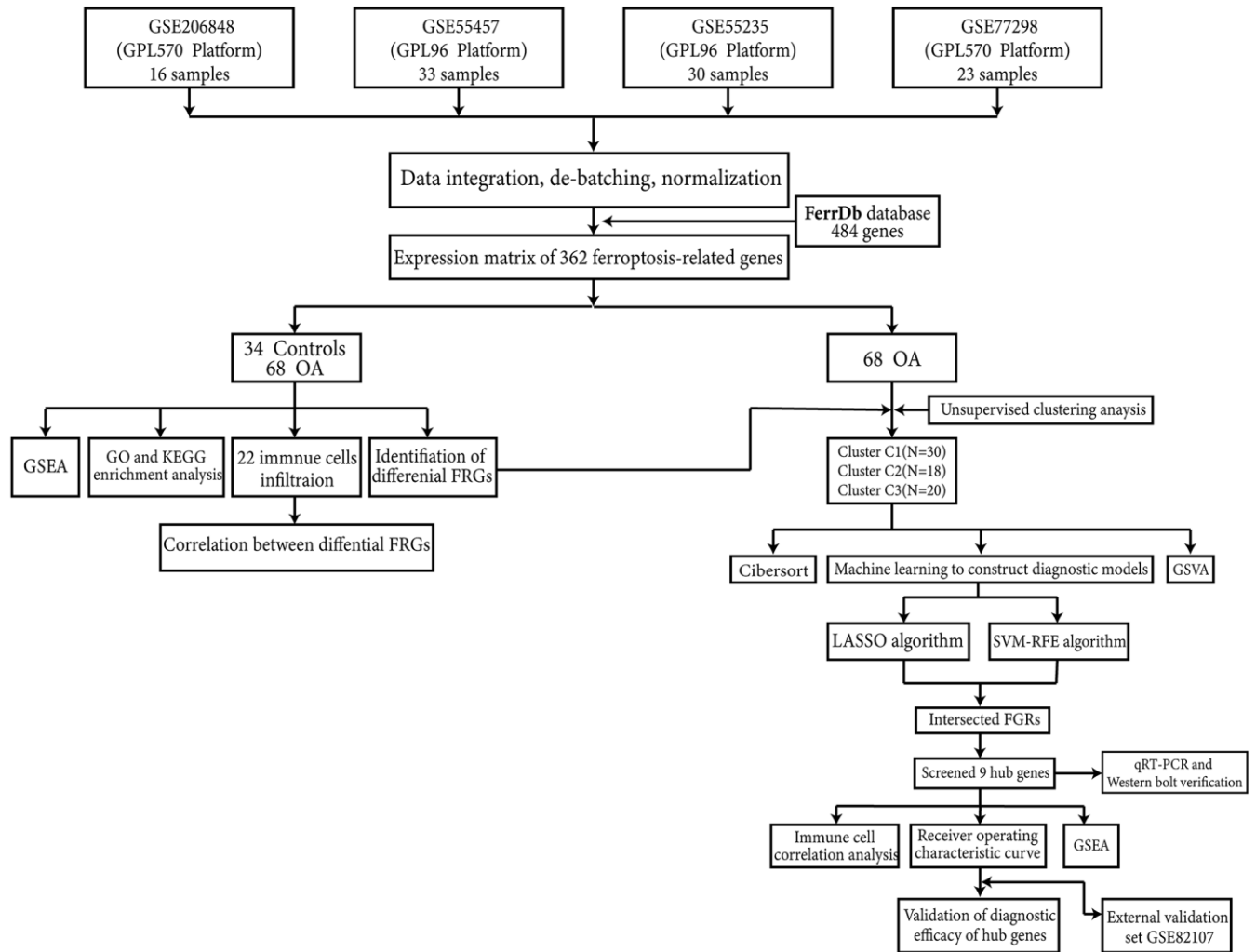

Supplementary Figure 1. The overall flow chart for this analysis.

Supplement: Supplementary Figures [file aging-16-205875-s001.pdf]
