# Supplementary material for: Machine learning-based identification and immune characterization of ferroptosis-related molecular clusters in osteoarthritis and validation
Source: Aging (Albany NY). 2024 May 29;16(11):9437–59. doi: 10.18632/aging.205875 (PMC11210262; doi:10.18632/aging.205875)
Supplement: Supplementary Tables [file aging-16-205875-s002.pdf]

SUPPLEMENTARY TABLES

Supplementary Table 1. Information on the training datasets.

Data number	Platform information	Osteoarthritis group	Control group	Species
GSE206848	GPL570	9	7	Homo sapiens
GSE55457	GPL96	23	10	Homo sapiens
GSE55235	GPL96	20	10	Homo sapiens
GSE77298	GPL570	16	7	Homo sapiens

Supplementary Table 2. Sequences of primers used for real-time PCR.

Gene symbol	Sequence (5´- 3´)
GABARAPL1-F	TTCTGGTTGCACAATACTGGATGCC
GABARAPL1-R	CCGGATAAATAACACCTTCTGCCCC
SAT1-F	AGAGGTGCTTCTGATCTGTCCAGTG
SAT1-R	ATGGAGGTTGTCATCTACAGCAGCA
EGFR-F	TGACTACCAGCAGGACTTCTTTCCC
EGFR-R	TCCTCCGTGGTCATGCTCCAATAAA
ELOVL5-F	GCACATTCCCTCTTGGTTGGTTGTA
ELOVL5-R	TGGTCCTTCAGGTGGTCTTTCCTTC
TBK1-F	AGTGGAATCAAACATACCATGACCCC
TBK1-R	AGAGCCAAACCTTTCTAAAATGTGGT
TRIM26-F	TGACTTTCACCAACGCAGAGTCACA
TRIM26-R	CAGATGTCAGGGCTCAGGGTCTTAG
SLC39A7-F	TGTGGGTTCTCAGTGGAATTGTTGC
SLC39A7-R	GAACGCTCTTGTCTTCCATGTCCATG
PTPN6-F	CAGAAGCAGGAGGTGAAGAACTTGC
PTPN6-R	GCTGTGGTCAAAGGGGAGAATGTTC
BEX1-F	TGGAGTCCAAAGAGAAACGAGCAGT
BEX1-R	TCTAGGCACACAGTATTCACCAGCA
MMP13-F	AACGCCAGACAAATGTGACCCTTC
MMP13-R	CGTTAAAAACAGCTCCGCATCAACC
Collagen II-F	ACCTTGGACGCCATGAAGGTTTTC
Collagen II-R	CTTGCTGCTCCACCAGTTCTTCTTG
GAPDH-F	GACATCAAGAAGGTGGTGAAGCAGG
GAPDH-R	GTGTCGCTGTTGAAGTCAGAGGAGA
