# Supplementary material for: Machine learning-based identification and immune characterization of ferroptosis-related molecular clusters in osteoarthritis and validation
Source: Aging (Albany NY). 2024 May 29;16(11):9437–59. doi: 10.18632/aging.205875 (PMC11210262; doi:10.18632/aging.205875)
Supplement: Supplementary File 1 [file aging-16-205875-s003.pdf]

Supplementary File

Supplementary File 1. (LASSO coefficients).

**GABARAPL1**	-6.55E-01
**SAT1**	-4.24E-01
**EGFR**	-4.00E-01
**ELOVL5**	-3.27E-01
**TBK1**	-6.40E-01
**SLC39A7**	-7.50E-01
**TRIM26**	6.44E-01
**PTPN6**	3.26E-01
**BEX1**	-6.41E-01
