# Supplementary material for: Machine learning-based identification and immune characterization of ferroptosis-related molecular clusters in osteoarthritis and validation
Source: Aging (Albany NY). 2024 May 29;16(11):9437–59. doi: 10.18632/aging.205875 (PMC11210262; doi:10.18632/aging.205875)
Supplement: Supplementary File 5 [file aging-16-205875-s007.pdf]

Supplementary File 5. Unsupervised clustering subtype distribution.

Sample	Group
GSM6265697_Treat	C1
GSM6265698_Treat	C1
GSM6265699_Treat	C3
GSM6265700_Treat	C1
GSM6265701_Treat	C2
GSM6265702_Treat	C1
GSM6265703_Treat	C2
GSM6265704_Treat	C3
GSM6265705_Treat	C1
GSM1337314_Treat	C3
GSM1337315_Treat	C3
GSM1337316_Treat	C1
GSM1337317_Treat	C1
GSM1337318_Treat	C2
GSM1337319_Treat	C2
GSM1337320_Treat	C1
GSM1337321_Treat	C1
GSM1337322_Treat	C1
GSM1337323_Treat	C1
GSM1337324_Treat	C1
GSM1337325_Treat	C1
GSM1337326_Treat	C1
GSM1337327_Treat	C3
GSM1337328_Treat	C3
GSM1337329_Treat	C2
GSM1337330_Treat	C3
GSM1337331_Treat	C3
GSM1337332_Treat	C3
GSM1337333_Treat	C3
GSM1337334_Treat	C3
GSM1337335_Treat	C3
GSM1337336_Treat	C2
GSM1332211_Treat	C2
GSM1332212_Treat	C3
GSM1332213_Treat	C2
GSM1332214_Treat	C2
GSM1332215_Treat	C2
GSM1332216_Treat	C3
GSM1332217_Treat	C1
GSM1332218_Treat	C1
GSM1332219_Treat	C3
GSM1332220_Treat	C2
GSM1332221_Treat	C1
GSM1332222_Treat	C1
GSM1332223_Treat	C1
GSM1332224_Treat	C1

GSM1332225_Treat	C1
GSM1332226_Treat	C1
GSM1332227_Treat	C1
GSM1332228_Treat	C1
GSM1332229_Treat	C1
GSM1332230_Treat	C1
GSM2048272_Treat	C2
GSM2048273_Treat	C1
GSM2048274_Treat	C1
GSM2048275_Treat	C3
GSM2048276_Treat	C1
GSM2048277_Treat	C3
GSM2048278_Treat	C3
GSM2048279_Treat	C1
GSM2048280_Treat	C2
GSM2048281_Treat	C2
GSM2048282_Treat	C3
GSM2048283_Treat	C2
GSM2048284_Treat	C3
GSM2048285_Treat	C2
GSM2048286_Treat	C2
GSM2048287_Treat	C2
